# Extraction of a Needle

**Published:** 1869-06

**Authors:** C. N. Cooper

**Affiliations:** Cresco, Iowa


ARTICLE XXIV.

### EXTRACTION OF A NEEDLE.

BY C. N. COOPER, M.D.

In May, 1868, Gertrude C., aged 2 years, was suddenly seized with violent coughs, choking, and dyspnœa, which lasted several hours, after which she breathed naturally. In November following, she complained that something hurt her back.

On examination, a foreign substance, apparently a small pin, was discovered just below the inferior angle of the right scapula, pointing externally. It disappeared and reappeared several times. *April 13th.* I was called, with Dr. Clemmer, of this city, to examine the child, and, if practicable, remove the offending substance. We cut down upon the point, seized with forceps, and extracted the pointed end of a needle, about eight lines in length.

There was no suppuration before nor after the operation. Is it probable that the needle passed down the larynx and worked its way through the tissues to the surface? Is it more probable that it was thrust into the tissues from without, unnoticed by the child and its attendants?

I should be glad to hear any suggestions on this point.

Cresco, Iowa, *April 27th, 1869.*

